# Initial meconium microbiome in Chinese neonates delivered naturally or by cesarean section

**DOI:** 10.1038/s41598-018-21657-7

**Published:** 2018-02-19

**Authors:** Yi-Chao Shi, He Guo, Jing Chen, Gang Sun, Rong-Rong Ren, Ming-Zhou Guo, Li-Hua Peng, Yun-Sheng Yang

**Affiliations:** 1 Department of Gastroenterology and Hepatology, Institute of Digestive Diseases, Chinese PLA General Hospital, Beijing, China; 2 Realbio Genomics Institute, Shanghai, 200050 China

## Abstract

Previous studies have revealed significant differences in microbiome compositions between infants delivered via cesarean section (C-section) and natural vaginal birth. However, the importance of the delivery mode in the first days of life remains unclear. Importantly, this stage is minimally affected by infant feeding. Here, we used a metagenomic sequencing technique to characterize the meconium microbiome from the feces of a Chinese cohort of vaginally and C-section-delivered infants, including *in vitro* fertilization (IVF) newborns, during the first 24 h after birth. Meconium microbiome diversity was higher in vaginally delivered infants than that in C-section-delivered infants. *Propionibacterium* species were most abundant in the vaginally delivered infants, whereas the C-section group had high levels of *Bacillus licheniformis*. The two IVF newborns delivered by C-section harbored microbial communities similar to the vaginal microbiome in terms of taxonomic composition. Metabolic functions of the C-section group suffered more from the influence of the dominant group (*B. licheniformis*), whereas the vaginal group was more homogeneous, with a metabolism dominated by multi-microbes. Moreover, different modes of delivery affected the antibiotic resistance gene (ARG) prevalence. These findings provide novel information for the development of strategies to guide a healthy mode of delivery and promote the formation of healthy microbiota.

## Introduction

The complex community of the gut microbiota plays key roles at different stages of human life, including the newborn phase^1^. Early colonization by the microbiota is the basis of its assistance with later physiological growth, immunological maturity and neurological homeostasis for adequate infant development. However, it remains unclear at what stages of fertilization, pregnancy or birth this process of microbiota establishment begins.

Analyses of microorganisms in human follicular fluid and endometria early during the fertilization period in women undergoing *in vitro* fertilization (IVF) have revealed the presence of bacterial species correlated with IVF pregnancy outcomes and embryo transfer rates^2,3^. During pregnancy, a unique low-abundance microbiome has been detected in placental tissue^4^, amniotic fluid^5^, umbilical cord blood^6^, and fetal membranes^7^ from healthy newborns without any indication of infection or inflammation. However, major microbial colonization of the gastrointestinal tract begins at birth, when an infant comes into contact with microbes from the extra-uterine environment^8,9^. This initial exposure of the fetus during pregnancy and delivery is assumed to lead to gradual compositional development and finally to the establishment of a stable, individual-specific microbiota^10^. The mode of delivery and subsequent environment exposures greatly influence the composition of the microbiota in the infant. These early colonization events and the establishment of the intestinal microbiota have an effect on the development of a variety of diseases later in life^11^. Compared with vaginal delivery, cesarean section (C-section) was demonstrated to correlate with increase in the incidence of diseases such as asthma^12^, allergic rhinitis, obesity^13^, celiac disease^14,15^ and type I diabetes^16^. The microbiota of infants delivered by C-section can be partially restored by re-exposure to maternal vaginal fluids at birth^17^. Therefore, identifying the pioneer environment established by the colonization of pioneer microbes may provide a new perspective for the prevention and treatment of disease.

A recent systematic reviewer by Rutayisire *et al*.^11^ indicated that C-sections were associated with a lower abundance and diversity of the phyla Actinobacteria and Bacteroidetes and a higher abundance and diversity of the phylum Firmicutes from birth to 3 months of life based on analyses using culture-dependent or culture-independent techniques (16 S rRNA gene sequencing/amplification, molecular methods or fluorescence *in situ* hybridization). At the taxonomic level, the genera *Bifidobacterium* and *Bacteroides* appear significantly more frequently in vaginally delivered infants than in C-section delivered infants. Conversely, these latter infants are colonized more by bacteria from the genera *Clostridium* and *Lactobacillus*. A study by Dominguez Bello *et al*.^8^ using 16 S rRNA sequencing demonstrated that the guts microbiota of vaginally delivered Amerindian infants resembled their mothers’ vaginal microbiota, which were dominated by *Lactobacillus*, *Prevotella*, or *Sneathia* spp.; in contrast, infants delivered by C-section were colonized by common skin and environmental microbes, such as *Staphylococcus*, *Corynebacterium*, and *Propionibacterium* spp. Gosalbes *et al*.^18^ employed 16 S rRNA sequencing to examine the meconium microbiota of Spanish infants and found that these meconium microbiota were dominated by lactic acid or enteric bacteria. Dong Liu *et al*.^19^ investigated the intestinal microbiota of Chinese newborn infants on days 2 and 4 of postnatal life and demonstrated that the dominant bacteria found in vaginally delivered infants were *Acinetobacter* spp., *Bifidobacterium* spp., and *Staphylococcus* spp., whereas *Citrobacter* spp., *E. coli*, and *Clostridium difficile* were more common in C-section delivered infants.

Diversity analyses of the microbiota in vaginal and C-section delivered infants are partially dependent on race or geographical differences^20^. Additionally, the interval of meconium sample collection varies from within 24 h after delivery to 4 days, which might contribute to differences in the microbiota with different mode of delivery^8,19,21^.

Here, we assessed the gut microbiota composition in the first-pass meconium of 8 vaginally and 10 C-section (including two IVF newborn)-delivered Chinese infants. We assembled gut microbial genomes and demonstrated gut microbiome signatures that are characteristic of early life. In conclusion, we identified the characteristics of the pioneer environmental pattern according to different modes of delivery, including both the composition and metabolic functions of the microbiota.

## Results

### Characteristics of the meconium microbiomes associated with different modes of delivery

To characterize the infant meconium microbiome, we performed metagenomic sequencing analysis and compared the microbial community structures between vaginally and C-section-delivered infants. As shown in Table 1, 16 of 18 infants (8 from vaginal delivery and 8 from C-section delivery) were born at term after a normal pregnancy at a gestational age of 37–42 weeks, and 2 of 18 infants (C-section) were born at a gestational age of 36–37 weeks after an IVF pregnancy. There were no significant differences in the mother’s age, mother’s height, mother’s gestational weight gain, birth weight or infant length between the two groups. The vaginal delivery group had higher gestational age days than the C-section group (*P* = 0.044). Patients with a higher pregnancy weight were more common in the vaginal delivery group.

**Table 1:** Clinical characteristics of the mothers and infants included in the study.

Variable	Vaginal delivery N = 8	C-section delivery N = 10	*P*
Mother’s age (years)	29 (23–38)	29.8 (25–33)	0.649
Mother’s height	164.5 (158–174)	163.5 (157–170)	0.664
Mother’s pre-pregnancy weight (kg)	69.6 (57–89)	80.8 (68–97)	0.050
Mother’s gestational weight gain (kg)	13.1 (7–20)	13.1 (9–17)	0.997
Gestational age (days)	280.6 (273–287)	271.0 (254–288)	0.044
Birth weight (gram)	3381.1 (3000–3900)	3565 (3100–3900)	0.189
Birth length (cm)	49.9 (49–53)	51.1 (49–53)	0.086
Baby gender (boy/girl)	0/8	5/5	0.036^#^

^#^Fisher’s exact test.

We obtained an average of 33.74 GB of raw data for each sample, with a range from 30.15 GB to 60.04 GB. After filtering out the low-quality reads and host contamination, we obtained 2.33 GB to 5.38 GB of clean data for each sample (average 3.62 GB).

The data yielded sequences belonging to 9 phyla and 141 species, including several DNA viruses (Fig. S1A,B) and a small amount of fungi (data not shown). Firmicutes was the most abundant phylum, accounting for 37.4% of the total reads, followed by Actinobacteria with 28.3%, Proteobacteria with 21.2%, unidentified viruses with 8.9%, Bacteroidetes with 3.7% and other phyla with 0.4% (Fig. S1C).

### Mode of delivery significantly affected the first-pass meconium microbiome

**Figure 1:**
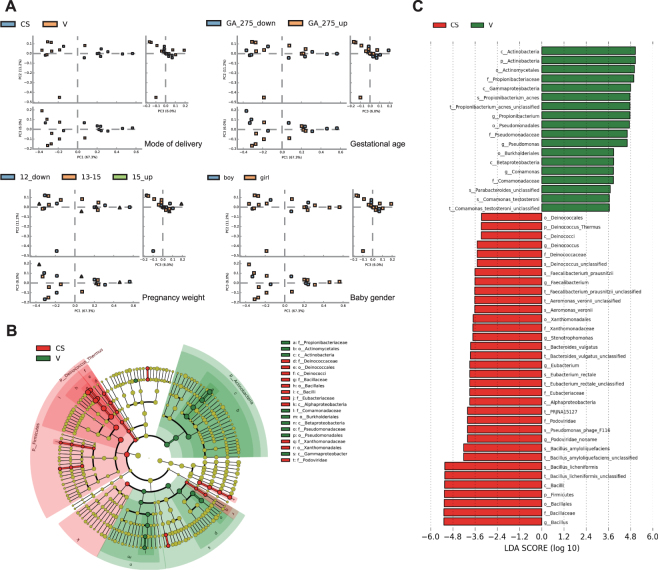
Comparison of the microbiomes of vaginally delivered and C-section-delivered newborns. (**A**) Principal-component analysis (PCA) plot based on the relative taxa abundance in the fecal microbiomes of neonates with mode of delivery, gestation age, pregnancy weight or gender groups. Sample are marked by the group type. The number is the grouping boundaries. (**B**) LEfSe comparison of microbiomes from meconium samples from vaginally delivered and C-section-delivered neonates. Enriched taxa in samples from vaginally delivered infants with different level classifications with a positive linear discriminant analysis (LDA) score are shown in green; C-section samples with a negative LDA score are shown in red (>3.5 in both cases). (**C**) Cladogram derived from LEfSe analysis of metagenomic sequences from C-section delivered newborn and vaginally delivered neonate meconium samples. Green shaded areas indicate microbe orders that more consistently describe the fecal microbiome from vaginally delivered infants; red shaded areas indicate microbe orders that more consistently describe the meconium microbiome from C-section-delivered infants. The prefixes “p”, “c”, “o”, “f”, “g”, “s”, and “t” represent the annotated level of phylum, class, order, family, genus, species and strain.

To illustrate the relationship between the microbial composition and metadata, redundancy analysis (RDA), multivariate ANOVA based on dissimilarities (Adonis) and principal component analysis (PCA) was performed using species data at all sample levels. Figures 1A and S2 revealed that the delivery modes were the most significant contributors to the composition of the newborn neonate gut microbiomes in contrast to other factors (Figs 1A, S2A,B and Table S1). The Simpson and Shannon-Weiner indices, which measure the alpha-diversity of the species richness and the evenness of distinct microbes in a community, respectively, were plotted and compared. The results indicated that the microbiome of the vaginally delivered infants was somewhat more species-rich and diverse than the microbiome of the C-section-delivered infants (Fig. S3). The metagenomic sequence data were equivalent at the species (strain) level classification. A LEfSe analysis and cladogram were performed to investigate differences in the community composition between groups (Fig. 1B,C). The phyla Firmicutes and Deinococcus-Thermus were significantly enriched in the vaginally delivered group, whereas Actinobacteria was higher in abundance in the C-section delivered group (Fig. S4). There were six significantly different classes, with enrichment of Actinobacteria, Gammaproteobacteria, and Betaproteobacteria in the vaginal group and Deinococcus, Alphaproteobacteria and Bacilli in the C-section delivered group (Fig. 1B,C). The microbial composition also significantly differed at the species level, with 10 significantly different species between groups. *Propionibacterium acnes*, *Parabacteroides* unclassified, and *Comamonas testosteroni* exhibited relatively higher abundance in the vaginally delivered group. *Bacillus licheniformis*, *Bacillus amyloliquefaciens*, *Eubacterium rectale*, *Bacteroides vulgatus*, *Aeromonas veronii*, *Faecalibacterium prausnitzii* and *Deinococcus* unclassified were relatively more abundant in the C-section-delivered group (Fig. 1B,C).

Additionally, we identified a number of viruses in the meconium. The bacteriophage families were the most prevalent DNA virus families detected in early life specimens. Further study using an extended error bar plot analysis found that the *Pseudomonas* phage F116 was more enriched in the C-section delivered neonate specimens (Fig. S5).

**Figure 2:**
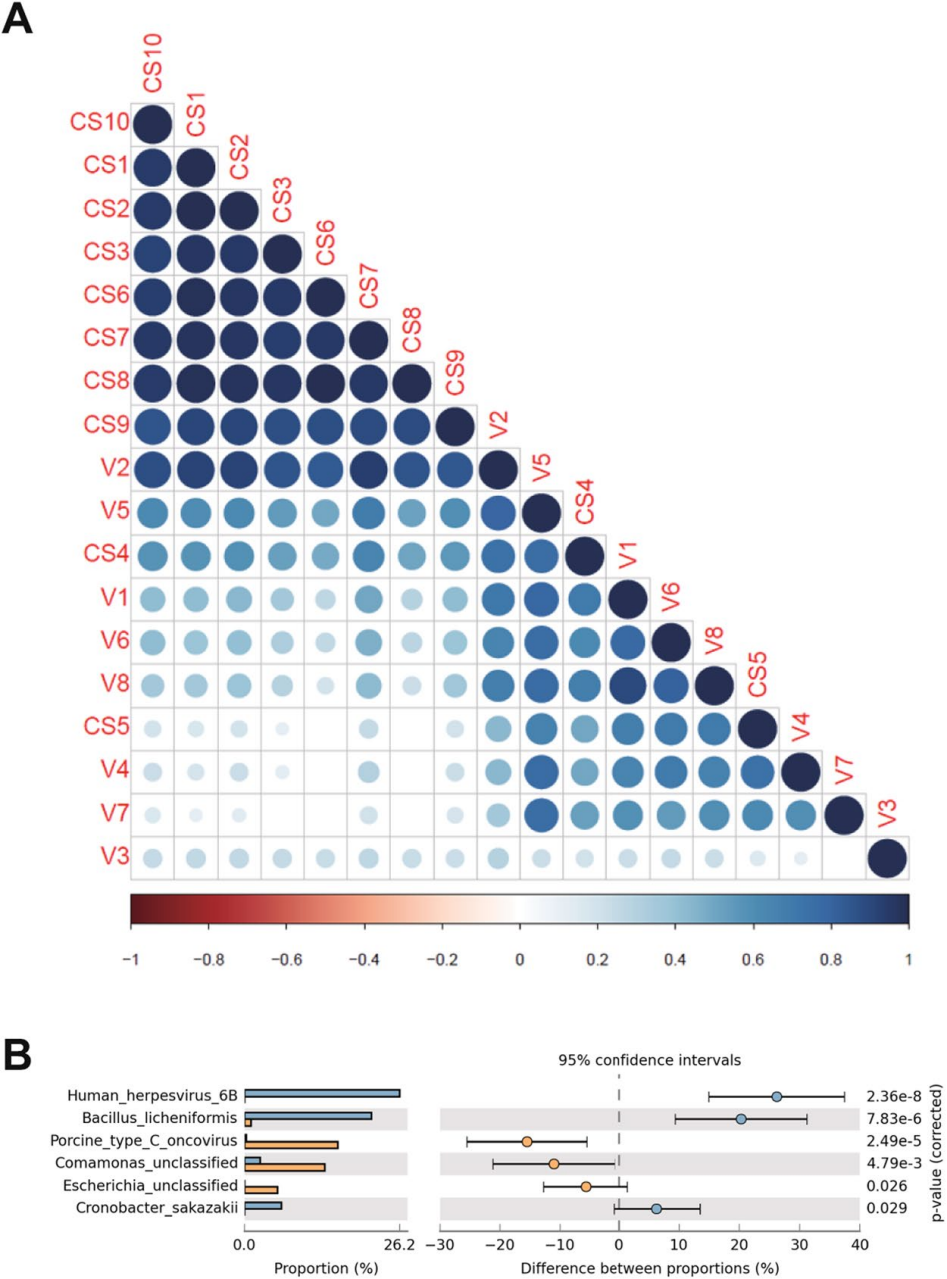
Spearman correlation heat map analysis showed that the fecal microbiomes from IVF infant samples were clustered with those from the vaginally delivered infant samples. (**A**) CS4 and CS5 samples represent IVF neonates whose fecal microbiomes clustered with those from the vaginally delivered infants. (**B**) Distribution of the relative abundance of taxa between the CS4 and CS5 IVF samples. Horizontal bars indicate 95% confidence intervals from the mean proportion.

The phylogenetic composition of the IVF newborn samples (CS4 and CS5) clustered similarly to the samples from the vaginally delivered fecal microbiomes (Fig. 2A). Additionally, the CS4 samples had an abundance of the human herpesvirus 6B, *B. licheniformis* and *Cronobacter sakazakii* microbes, whereas CS5 were richer in *Comamonas* unclassified, porcine type-C oncovirus and *Escherichia* unclassified (Fig. 2B). Other samples showed distinct phylogenetic compositions between the vaginal and C-section delivery groups using Bray Curtis cluster analysis.

### Correlation of the predominant microbiome with different modes of delivery

**Figure 3:**
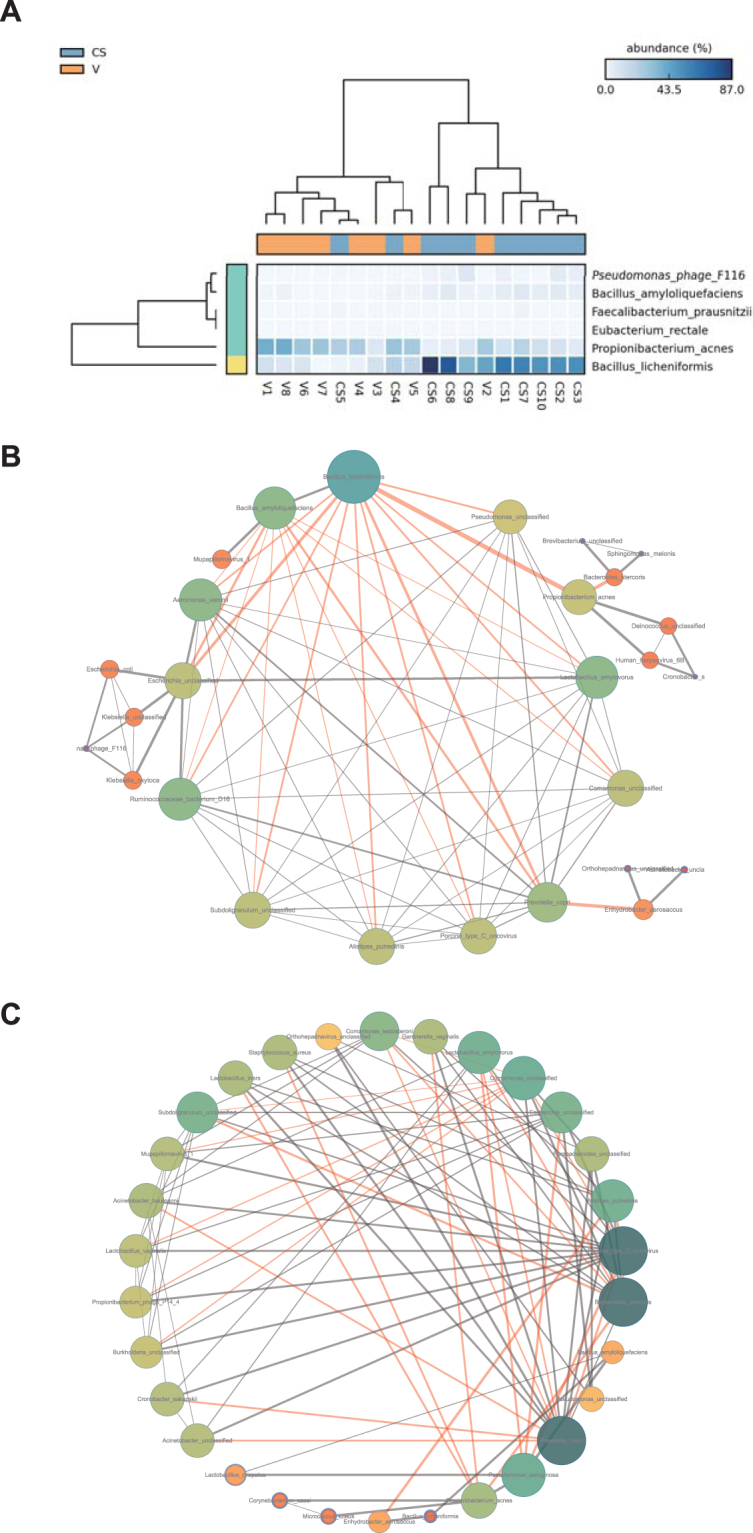
Correlation of the predominant microbiomes under different modes of delivery. (**A**) Heat map analysis generated with LEfSe showing the taxonomic strains with significantly different abundance in C-section and vaginally delivered neonates. The dendrogram of pathway abundance is colored red to indicate a negative LDA score and green to indicate a positive LDA score. Over/underrepresentation is depicted by a color gradient. (**B**,**C**) Correlations were expressed by Spearman’s correlation coefficients. Only the first 30 abundant species were analyzed. Network analysis of C-section-delivered infant meconium microbes (**B**) and vaginally delivered infant meconium microbes (**C**). Nodes indicate taxonomic affiliations at the species level. Red lines indicate negative correlations; gray lines indicate positive correlations. The line thickness represents the correlation strength. The size of a node is determined by the thickness of all lines on the node.

Extended error bar plots and associated confidence intervals with bootstrapping were constructed to determine the mean differences for each species between the vaginally and C-section-delivered fecal samples (Fig. 3A). Compared with the vaginal delivery samples, the C-section group contained significantly higher levels of *B. licheniformis* and *B. amyloliquefaciens* at the species level (P < 0.05) (Fig. 3A). The relative abundance of *P. acnes* was higher in the vaginal delivery group than in the C-section delivered samples. Both the C-section- and vaginally delivered infants showed a relatively low abundance of *Lactobacillus*. However, due to the relative lack of lactobacilli in the C-section infants, the diversity and abundance of lactobacilli in the vaginal abundance and delivery samples were still higher than the abundance in the C-section group (Fig. S6).

Recently, research has shown that the neonate intestinal microbiota is significantly affected by the mother’s microbiome. The differences in the mode of delivery may affect the fecal, vaginal, skin, mouth and environmental microbiota and, consequently, the establishment of the pioneer environment. The mode of delivery affected taxonomies and consistencies across individuals, demonstrating that a pioneer environmental pattern could directly affect the colonization of the gut microbiome during early life. We focused on the different predominant microbiomes based on the mode of delivery and explored the relationships among the microbes using a network analysis. Figure 3B,C depict a complex network-based analysis of the meconium microbiome obtained using the Cytoscape program. This analysis revealed that the microbial communities in the C-section group were dominated by inhibitory relationships. Among them, *B. licheniformis* displayed the strongest inhibitory relationship to the surrounding microbes in the cluster. In the vaginally delivered group, the interactions between microbial communities were higher, with larger correlations that were both positive and negative. *Bacillus* was associated with *Pseudomonas* and *P. acnes* in terms of connections with other microbe clusters and was not a major factor in the relationships between the entire microbiota of the C-section group and that of the vaginally delivered group.

### Functions of metabolism in the meconium microbiome in vaginally and C-section delivered neonates

**Figure 4:**
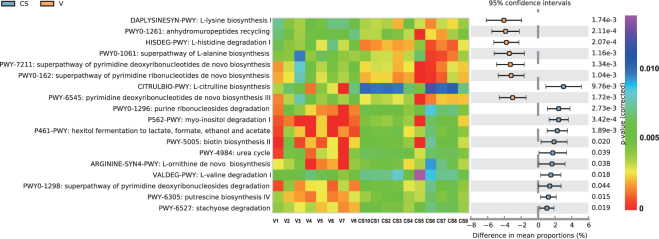
Extended error bar plots showing functional properties that differ between C-section and vaginally delivered neonate gut microbiomes. Species identified by LEfSe as different between the two groups are depicted. The proportion (left side) indicates the possible abundance of microbes possessing each functional feature and the difference between proportions for each feature. For this analysis, features were filtered by the q value (0.05) and effect size (0.01). Blue indicates the C-section group; orange indicates the vaginal group.

To detect changes in microbiome metabolites induced by changes in the microbiota abundance, the metagenomics data were annotated with metabolic pathways from the MetaCyc database using the HumanN2 program. According to the LEfSe analysis heatmap of the metabolic function pathways, most differences occurred in amino acid metabolism pathways. The meconium microbiome of the vaginally delivered infants included more relative amino acid biosynthesis and amino acid degradation than did the C-section microbiome. Nucleoside and nucleotide biosynthesis was more abundant in the vaginally delivered infants, whereas their degradation was elevated in the C-section infants. Moreover, carbohydrate biosynthesis, inorganic nutrient metabolism, acetyl-CoA biosynthesis, and amine and polyamine metabolism were more active in the C-section delivered newborns. Consistent with the taxonomic composition according to the mode of delivery, PCA also showed a significant difference in pathways between the C-section and vaginal groups (Fig. 4).

### Influence of different modes of delivery on the ARG prevalence

**Figure 5:**
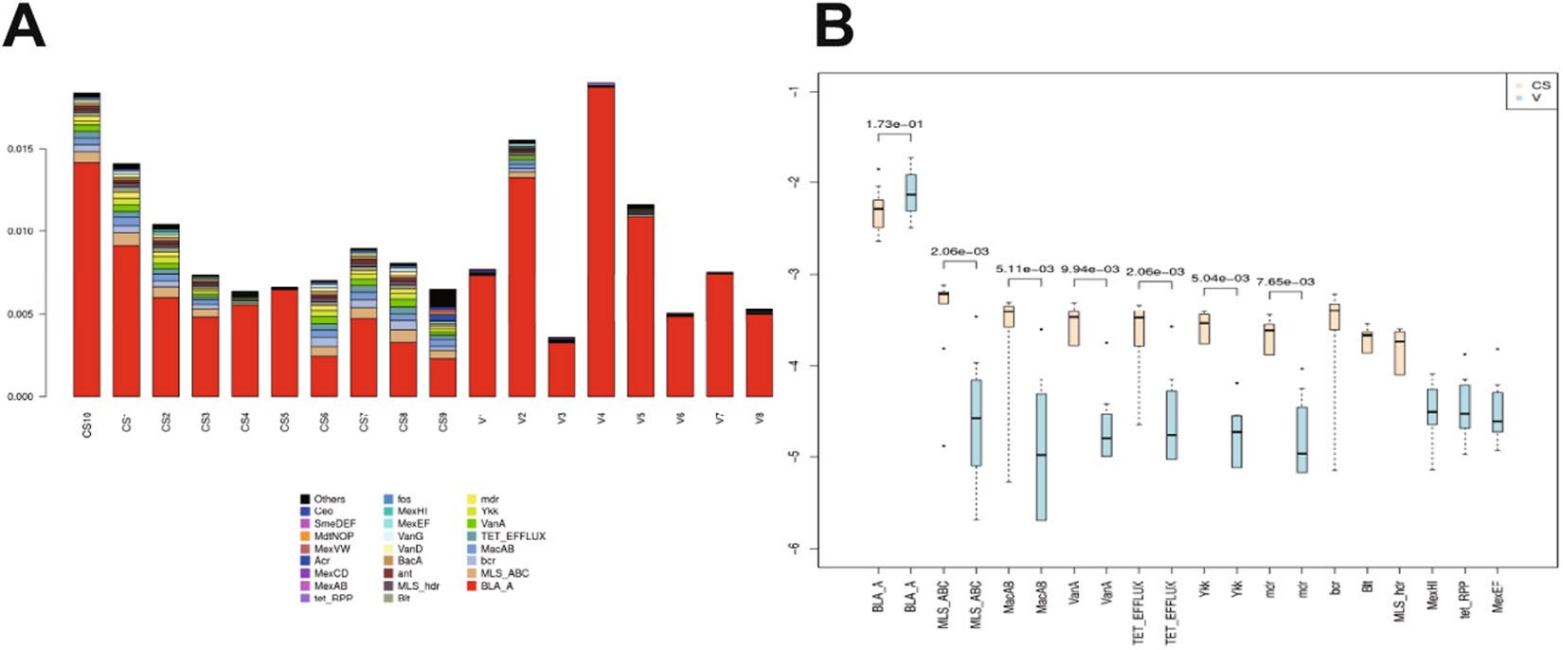
Influence of different modes of delivery on the prevalence of ARG. (**A**) Prevalence of antibiotic resistance genes in the meconium microbiome in neonates. (**B**) Correlation of antibiotic resistance genes with different modes of delivery. *P < 0.05.

We quantified the relative abundance of ARGs in the meconium DNA to characterize the pioneer gut microbiome environment. The top 15 main ARG classes in each sample are shown in Fig. 5A. The *BLA_A* showed significant abundance in the meconium microbiome and enrichment in the vaginally delivered neonates. *MexHI*, *tet_RPP* and *MexEF* were more represented in the vaginally delivered neonates than in the C-section-delivered counterparts. Neonates delivered by C-section showed a significantly higher prevalence of *MLS_ABC*, *MacAB*, *VanA*, *TET_EFFLUX*, *Ykk*, *mdr*, *bcr*, *Blt* and *MLS_hdr* than the vaginally born neonates (Fig. 5B).

### Correlation between metabolism and microbiome in different modes of delivery

To understand the correlation between microbiota community structure differences and metabolic differences between the vaginally delivered group and the C-section delivered group, we conducted a correlation analysis between the two groups. The results revealed a closer relationship between microbes in the vaginal group.

**Figure 6:**
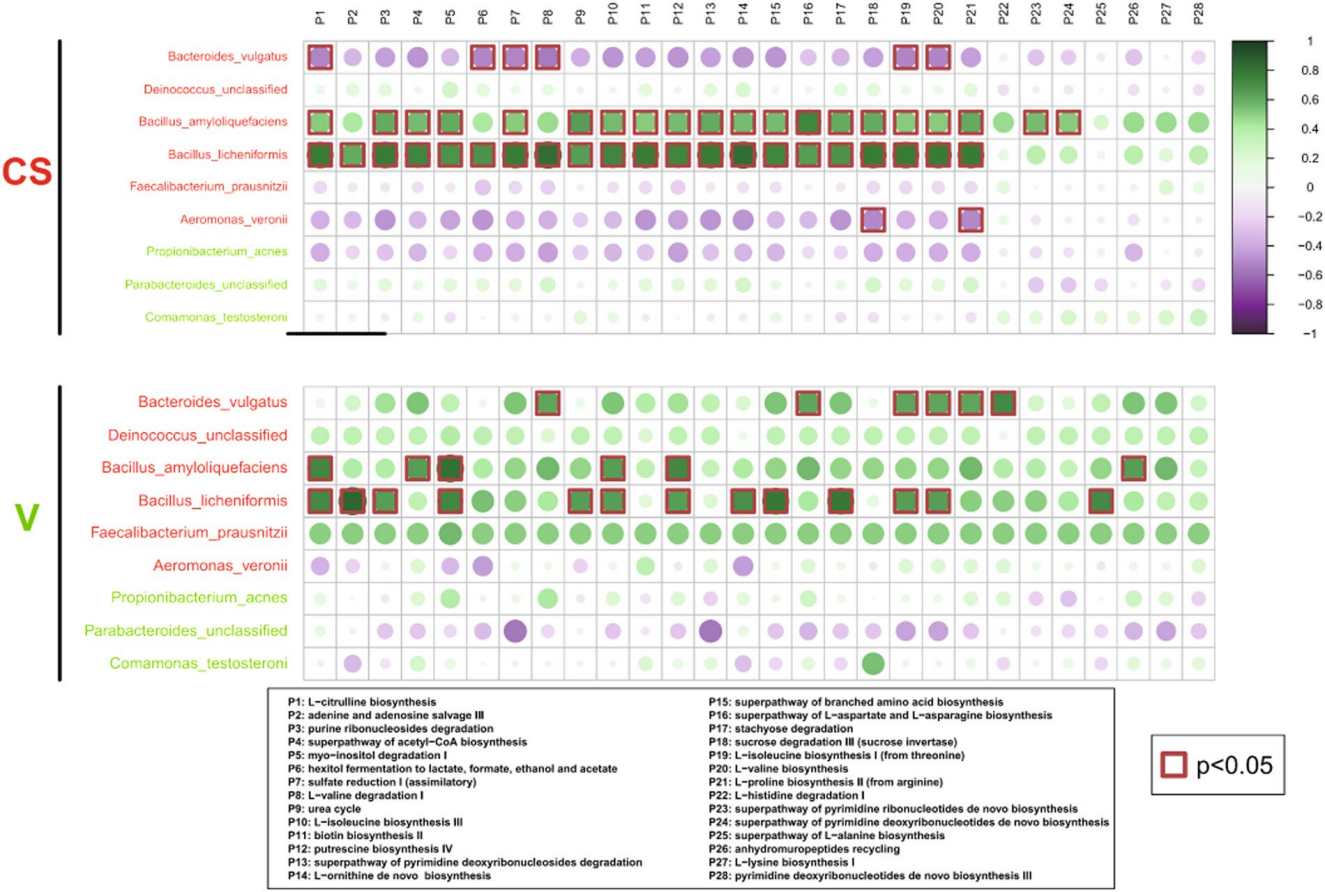
Microbial metabolic differences in the meconium microbiome between vaginally delivered and C-section-delivered neonate samples. Genera identified by LEfSe as different between the two biological samples are depicted. Metabolic pathway designations are delineated at the bottom of the figure. The shading intensity and size of the circles indicate the Kendall rank correlation coefficient between matrices. Negative correlations are shown in violet, and positive correlations are shown in blue. Red squares surrounding the circles are indicative of a *P* value ≤ 0.05.

As shown in Fig. 6, the abundance of *B. licheniformis* and *B. amyloliquefaciens* in the C-section group had a significant positive effect on the metabolic pathway abundance; almost all other species showed a negative correlation. The abundance of these metabolic pathways was affected by multiple microbiota in addition to *B. licheniformis* in the vaginal group. The diversity of the microbial composition in vaginal delivery involved in the newborn intestinal microbial metabolism was also higher, which helped avoid the emergence of certain types of metabolic deficiencies. Because a greater variety of microorganisms were present in the vaginal group, the metabolic pathways were more abundant and diversified (Fig. S7A). The diversity index is a quantitative measure that reflects the number of different types in a dataset. The Shannon index refers to the amount of the difference, whereas the Pielou index refers to the degree of closeness. Thus, the Shannon and Pielou indices of the metabolic pathways were also calculated. Although there was no difference in metabolic pathway diversity between the two groups in the Shannon index, the vaginal group exhibited more homogeneity in metabolic pathways in the Pielou index analysis (Fig. S7B). Together, these results indicate that the C-section group suffers more from the influence of the dominant group (*B. licheniformis*), whereas the vaginal group is more homogeneous in its metabolism, which is dominated by multi-microbes.

## Discussion

Colonization by pioneer microbes and gradual diversification in the infant intestinal tract play important roles in the establishment of a symbiotic system of interactions between the host and microbes^22^. Different modes of delivery may affect the colonization of pioneer microbes, which may be the first group of microbes that infants encounter as they pass through the birth canal or are delivered by C-section. We used metagenomic sequencing to detect differences in the neonatal meconium between vaginally and C-section delivered neonates. The results demonstrated that the composition of the meconium microbiome was significantly affected by the mode of delivery. The predominant microbes were Firmicutes, Actinobacteria and Proteobacteria at the phylum level in our study, which was consistent with previous studies^23,24^. We also found that the predominant species in the vaginal neonatal meconium was *Propionibacterium*, whereas the dominant species was *B. licheniformis* in the C-section group. Moreover, we report for the first time that the characteristic gut microbiota in two IVF neonates differed from that of other C-section neonates and more closely resembled that of the vaginally delivered infants.

The pioneer bacteria that colonize the postnatal intestinal tract contribute to the establishment of host-microbe interactions essential for optimal symbiosis, which is essential for health throughout life^10,25^. It has been reported that the infant microbiota undergoes substantial reorganization within the first 6 weeks of life in a manner that is driven not by mode of delivery but by body site^26^. That investigation found that there was no significant clustering in the neonatal meconium microbiota by mode of delivery^26^. Nevertheless, several studies have demonstrated that the neonatal intestine is rapidly colonized by maternal and environmental bacteria at birth and is strongly affected by the delivery mode^8,21,24,27–29^. It was recently reported finding that the fecal microbiota of vaginally delivered infants had significant enrichment of *Bacteroides*, *Parabacteroides* and *Megamonas*, whereas that of cesarean delivered infants had enrichment of *Prevotella*, *Streptococcus* and *Trabulsiella* in both neonates and 2-month-old Chinese infants^30^. Moreover, alterations in the microbiota composition and the effect of the delivery mode may be associated with many diseases, including metabolic diseases (i.e., obesity) and immune-related diseases (i.e., inflammatory bowel disease, asthma, and allergies)^10^. Therefore, clarifying the intestinal microbiome habitat of the pioneer environmental pattern during the early life of healthy neonates is very important for the prevention and treatment of related diseases.

Our metagenomic analyses showed that *Propionibacterium* was the predominant bacterium in vaginally delivered infants. Additionally, *Propionibacterium* was widely and closely cross-linked with other bacteria. A review by Sabrina Tamburini *et al*.^31^ suggested that *Propionibacterium* was the main group observed in C-sections; the authors reported that these bacteria were sourced from the common skin and environment microbiota and could even be detected at early stages in fetal umbilical cord blood. However, in a recent study by Fredrik Backhed *et al*.^24^, *Propionibacterium* was enriched in the meconium from the vaginal group based on a metagenomic analysis. *Propionibacterium* are dominant in skin flora and have also been reported to be present in the adult gut microbiota. Therefore, *Propionibacterium* in the meconium may be derived from skin or fecal microbes through contact during vaginal delivery.

We also observed that *B. licheniformis* was significantly abundant in the C-section infants. Because there is little contact with the mother during childbirth by C-section, the neonatal gut microbiota cluster is relatively isolated. These relatively isolated microbial clusters may have the same origin; for instance, *P. acnes* originates from the skin^32^, *Enhydrobacter aerosaccus* originates from the air, and *Prevotella copri*^33^ and *Escherichia* originate from the intestinal microbiota. However, the *Lactobacillus* cluster representing vaginal microbes is lacking, and the *B. licheniformis*-based cluster is not a common microbial component from the mother; instead, this cluster most likely originates from the environment. *B. licheniformis* is an aerobic, spore-forming, Gram-positive rod that can be isolated from a number of hospital and community environments, including water, air, soil, dust and wet surfaces^34^. Survival in these adverse conditions is characteristic of this bacillus species and is partially due to the formation of resistant endospores. These bacteria are usually regarded as nonpathogenic organisms or as simple contaminants. Although *B. licheniformis* was previously found in vaginal and adult feces, it has not been reported in the meconium. Because each area has its own unique environmental factors, the usefulness of the metagenome in in-depth studies of the meconium is small, especially for first day samples. The intestinal flora may also show significant changes with an increase in dietary factors. Therefore, our study found that environmental factors had a significant effect on the early microbiota in newborns delivered by C-section, which helped us better understand the correlational relationship with potential diseases.

Recently, viral microbiomes were also shown to play important roles in human health. Lim ES *et al*.^35^ demonstrated that the eukaryotic virome and the bacterial microbiome expanded in concert with a contraction of and shift in the bacteriophage virome composition from birth to 2 years of age. Our results also showed that bacteriophages were more abundant in early life. Additionally, there were significant differences in the pathogenic virus distribution, which was consistent with the process of delivery. The mode of delivery, which led to different bacteriophage compositions, might control the number of microbes operating under a “predator-prey” model, as described by Lotka-Volterra. Therefore, further research on the pioneer gut microbiological environment should also consider viral impacts, especially from bacteriophages.

The presence of microbes in the genitourinary system has an important effect on reproduction^36^, especially *in vitro* fertilization^2,3^. Some studies have investigated the presence of *Lactobacillus* spp. in ovarian follicular fluids, which are associated with embryo maturation and transfer. Other studies found that the *Lactobacillus*-dominated endometrial microbiota was associated with positive reproductive outcomes in IVF patients^37^. However, the effect of IVF on the microbiota of C-section newborns is not clear. In our study, we compared the microbiome composition of the two C-section-delivered IVF patients to that of the vaginally delivered group and found similarities.

The intestinal microbiomes can be altered by many factors, such as the mode of delivery, diet and drugs; its metabolic capabilities are altered accordingly and affect host health. Thus, it is critical to not only identify the types of bacteria that compose the intestinal microbiota but also understand their ability to participate in metabolic functions. Many studies have shown that intestinal microbial genes that do not include carbohydrate metabolism enzymes, such as glycoside hydrolase and polysaccharide lyase, cannot contribute to the degradation or metabolism of these materials by the body. These enzymes are particularly important for neonatal intestinal development. C-section deliveries are influenced by the dominant group (*B. licheniformis*). The lack of metabolic variety and the basal metabolism of a single type of *Bacillus* would more easily lead to dysbiosis once disturbed. In the vaginal group, the metabolic pathways were more abundant and diversified, and a variety of microorganisms were involved in each pathway. More aerobic metabolic pathways were enriched in the vaginal group, suggesting that environmental factors had a large influence during the vaginal delivery process.

The gut microbiota are currently considered reservoirs of ARGs with the potential for horizontal transfer to pathogenic species^38^. Additionally, several resistance genes, such as the *bla* genes, originate from environmental bacteria and pose major problems in clinical treatment^39^. The human gut microbiome harbors numerous functional ARGs that are influenced by many factors, such as the mode of delivery, duration of breastfeeding and number of siblings of the infant^40^. However, few studies have investigated the presence of ARGs in the meconium. Alicea-Serrano *et al*. tested for the presence of four *Tcr* genes [*tet(M)*, *tet(O)*, *tet(Q)* and *tet(W)*] in rectal swab samples from 10 infants taken after meconium passage and only detected *tet(W)* in one sample^41^. M. J. Gosalbes *et al*.^38^ revealed a high prevalence of *BLr* and *Tcr* in both meconium and early fecal samples. Here, we compared our sequencing data with the Antibiotic Resistance Genes Database (http://ardb.cbcb.umd.edu/) and found that the *BLA_A* genes were the most abundant ARGs in the meconium. Furthermore, we found that the C-section delivered meconium microbiome contained more ARGs than the vaginally delivered group in terms of both diversity and abundance, which might influence the infant’s health in later life through the dissemination of other potentially harmful bacteria in the gut.

Our results demonstrated that the microbiome and metabolic diversity of vaginally delivered infants were significantly higher than the corresponding factors in the C-section group. The predominant bacteria were *Propionibacterium* in the vaginally delivered infants and *B. licheniformis* in the C-section group. This dominant distribution of *B. licheniformis* in the gut microbiome of the C-section delivered newborns may inhibit the diversity of the overall microbiome. In contrast, long-term contact with the birth canal and access to the distribution of maternal microbes in the gut microbiomes of vaginally delivered neonates may inhibit the excessive growth of *Bacillus* bacteria. Therefore, vaginally delivered newborns will have more diversified species, resulting in more homogeneous metabolism.

It has been reported that significant differences in gestational age, maternal pregnancy weight gain and gender between groups can influence the microbiome^21,29,42^. In this study, with a relative limited sample size, the impact of these factors was not concerned. Despite this, we were able to unambiguously identify the delivery mode as the most significant factor contributing to the difference between the meconium samples. Of course, larger studies are warranted to validate our findings and further studies are needed to investigate the long-term influence of delivery mode on microbiome and infant health.

## Methods

### Sample collection and informed consent

The initial meconium samples were collected by the Department of Gynecology and Obstetrics of the Chinese PLA General Hospital from April 2012 to November 2012. All mothers enrolled in the study gave their informed consent. The collected clinical data included the gestational age, gestational weight and weight gain of the mother, mode of delivery, birth weight, and body length of the infant. None of the mothers who delivered vaginally received antibiotics during delivery. Each mother who delivered by C-sectionreceived prophylactic antibiotics (metronidazole 0.5 g iv. bid, and cefmetazole 1 g iv. bid, or amoxicillin clavulanate potassium 3 g iv. bid) after the child was delivered. None of infants received antibiotic treatment. Ten infants in this study were born via C-section delivery, of which 8 infants were from healthy term pregnancies (gestational age 267 to 288 days) and 2 infants were fertilized using IVF (gestational age 254 days). Among the C-section delivered neonates, that mode of delivery was chosen because of breech/abnormal fetal position (n = 5), uterine scarring (n = 2), or the pregnant women’s request (n = 3). Eight infants were born with healthy term pregnancies via vaginal delivery (gestational age 273 to 287 days). The exclusion criteria were infants with a low birth weight (<2500 g) and infants who received postnatal antibiotics or whose mothers used antibiotics before C-section. All neonates were transferred to the postpartum room within 30 min after delivery and were breastfed with some colostrum after delivery. Internal portions of the first-pass meconium stools from within 24 h of delivery were collected from sterile single-use diapers into sterile single-use collection tubes, and then frozen at −80 °C within 30 min of collection.

### Ethics statement

This study was undertaken with the approval of the Chinese PLA General Hospital Ethics Service Committee (2012–027). All experiments were carried out in accordance with the approved guidelines. Informed consent was obtained from all the parents of each child prior to sampling.

### DNA extraction and metagenomic sequencing

In the laboratory, the meconium was divided into 5 aliquots (each 200-mg) and immediately stored at −80 °C. A frozen aliquot (200 mg) of each fecal sample was processed using the QIAamp DNA Stool Mini Kit (QIAGEN, Hilden, Germany) for DNA extraction as previously described^43^. The DNA concentration was measured with a NanoDrop (Thermo Scientific), and the molecular size was estimated by agarose gel electrophoresis.

Metagenomic DNA libraries were constructed with 0.2 μg of genomic DNA according to the Illumina TruSeq DNA Sample Prep v2 Guide, with an average insert size of 500 bp. The quality of all libraries was evaluated using an Agilent Bioanalyzer with a DNA LabChip 1000 kit. Negative controls (sterile water) were included for all the experimental process and showed no amplification in the final DNA library. Sequencing was performed using an Illumina Hiseq2500.

### Quality control of Illumina HiSeq. 2500 reads

Illumina raw reads were subject to the following treatments: (1) reads with more than 3 ambiguous N bases were removed; (2) reads with less than 60% high-quality bases (Phred score ≥20) were deleted; and (3) the 3′ ends of the reads were trimmed to the first high-quality base. The subsequent high-quality reads were further mapped to some cereal genomes (*Oryza sativa*, Beijing Rice indica and *Triticum aestivum*) using SOAPaligner (version 2.21); any hit associated with the reads and their mated reads was removed. These filtered reads were admitted to the next steps of the analysis.

### De novo assembly of the Illumina short reads

SOAPdenovo (version 2.04), which is based on DeBrujin graph construction, was employed to assemble short reads with the parameters ‘-M 3 -u -L 100 -d 1 -F’. The k-mers (varying from 39 to 59 by 4) from each sample were tested. The resulting scaffolds were cut into contigs at ambiguous Ns, and only contigs longer than 500 bp were retained. The N50 was calculated for contigs of different k-mers, and only contigs with the largest N50 assemblies were attributed to the sample. All contigs were applied for gene prediction.

### Taxonomic and gene profiling

The microbial composition at each taxonomic level was defined using the MetaPhlAn2 program with default parameters. The program is available at http://huttenhower.sph.harvard.edu/metaphlan.

The relative gene abundance was determined with the procedure introduced by Qin N *et al*.^44^. When calculating the abundance of genes, the high-quality reads from each sample were aligned against the gene catalog using SOAPalign2.21 with the parameters ‘-r 2 -m 100 -x 1000′; only paired-end reads that could be mapped to the same gene were accepted. The antibiotic resistance genes (ARGs) were annotated against the ARG database using the same method.

### Pathway analysis

MetaCyc is a curated database of experimentally elucidated metabolic pathways from all domains of life. The database contains 2453 pathways from 2788 different organisms and is currently the largest available curated collection of metabolic pathways. The HUMAnN2 program can efficiently and accurately determine the presence, absence, and abundance of metabolic pathways in a microbial community based on metagenomic sequencing data. The MetaCyc metabolic pathway modules for imputed genes were assigned using the HUMAnN2 program with default parameters (prescreen-threshold = 0.01, identity-threshold = 0.50). The methodology is available at http://huttenhower.sph.harvard.edu/humann, and the program can be installed from PyPI (https://pypi.python.org/pypi/humann2) as a source code from the HUMAnN2 Bitbucket repository (https://bitbucket.org/biobakery/humann2/wiki/Home).

### Statistical analysis

The STAMP program is a statistical/econometric software system for time-series models with unobserved components, such as trends (available at http://stamp-software.com/). Using the program, we generated extended error bar (EEB) plots to show that some properties differed significantly between C-section and natural vaginal births with some filter parameters (ratio of effect proportion ≥2, difference between proportion ≥1, and *P* value = 0.05).

The LEfSe (linear discriminant analysis effect size) algorithm determines the features most likely to explain differences between classes by coupling standard tests for statistical significance with additional tests encoding biological consistency and effect relevance (available at http://huttenhower.sph.harvard.edu/galaxy)^45^.

R is a software environment for statistical computing and graphics, and Cytoscape is a software platform for visualizing complex networks and integrating them with any type of attribute data. Spearman’s correlation coefficient was calculated using R and visualized as a network using Cytoscape. These programs are available at https://www.r-project.org/ and http://www.cytoscape.org/, respectively. Redundancy analysis (RDA) and Adonis was performed to identify the relationship between the microbial composition and metadata with R program.

## Electronic supplementary material

Supplement Figure legends and table
