# Supplementary material for: Initial meconium microbiome in Chinese neonates delivered naturally or by cesarean section
Source: Sci Rep. 2018 Feb 19;8:3255. doi: 10.1038/s41598-018-21657-7 (PMC5818670; doi:10.1038/s41598-018-21657-7)

Initial meconium microbiome in Chinese neonates delivered naturally or by cesarean section

Author: Yi-Chao Shi, PhD[1*], He Guo, MD[1]*, Jing Chen, PhD[2]*, Gang Sun, PhD[1], Rong-Rong Ren, PhD[1], Ming-Zhou Guo, PhD[1], Li-Hua Peng, MD[1#], Yun-Sheng Yang, MD[1#]

[1]Department of Gastroenterology and Hepatology, Institute of Digestive Diseases, Chinese PLA General Hospital, Beijing, China.

[2]Realbio Genomics Institute, Shanghai, 200050, China

Supplement Figure legends S1-7 and Table 1:

Fig. S1. Association analysis of microbial components and metadata. A, B. Result of RDA and adonis. Mode of delivery is the most significant contributors for microbial composition whether in RDA1 (82.14%) or R^2 (explained variance).

Fig. S2. Microbial phyla and special taxonomies in the meconium based on different modes of delivery of neonates. A, Relative abundance of microbes at the phylum level based on shot-gun metagenomic sequences. B, Species-level data per sample are shown as a percentage of the total sequences in vaginally delivered and C-section delivered neonate meconium microbiomes. C, Percentages of the total phylum taxonomies are shown in a pie chart.

Fig. S3. Alpha diversity indices as indicators of microbiome community biodiversity of the meconium between vaginally delivered and C-section delivered neonates. The Simpson index, Shannon index, and Pielou index are shown as estimators.

Fig. S4. Comparison of microbiome community compositions at the phylum level for vaginally delivered infants and C-section delivered neonates. A, Firmicutes showed significant differences between the two groups. B, Actinobacteria showed significant differences between the two groups (P<0.05).

Fig. S5. Composition of the viral microbiomes with different modes of delivery. Extended error bar plots showing the relative abundance of microbes at the species level in all specimens. For this analysis, features were filtered by the *P* value (0.05) and effect size (0.05).

Fig. S6. Comparison of the *Lactobacillus* composition between vaginally delivered infants and C-section delivered neonates.

Fig. S7. Comparison of the microbiome metabolic biodiversity of vaginally delivered and C-section-delivered newborns. A, PCA plot based on the metabolism abundance and diverse in the fecal microbiomes of C-section- and vaginally delivered neonates. Each symbol represents a sample. B, Alpha diversity indices as indicators of microbiome metabolic biodiversity of the meconium between vaginally delivered and C-section-delivered neonates. The Shannon index and Pielou index are shown as estimators.

Supplement table 1. Statistical significance between the microbial composition and metadata was tested with multivariate ANOVA based on dissimilarities (Adonis).

	Df	SumsOfSqs	MeanSqs	F.Model	R2	Pr (>F)
weight_gain	2	0.1644	0.0822	0.8464	0.05857	0.5471509
gestational_age	1	0.30089	0.30089	3.0981	0.10719	0.0383206*
gender	1	0.40139	0.40139	4.1329	0.143	0.011996*
mode of delivery	1	0.83164	0.83164	8.5629	0.29627	0.0006664***
weight_gain:gestational_age	2	0.40101	0.2005	2.0645	0.14286	0.0756415
weight_gain:gender	1	0.07688	0.07688	0.7916	0.02739	0.5408197
weight_gain:mode of delivery	1	0.06318	0.06318	0.6505	0.02251	0.631123
gestational_age:mode of delivery	1	0.08202	0.08202	0.8445	0.02922	0.4705098
Residuals	5	0.4856	0.09712	0.173		
Total	15	2.807	1			

**P*<0.05; ****P*<0.001

Supplement Figure 1

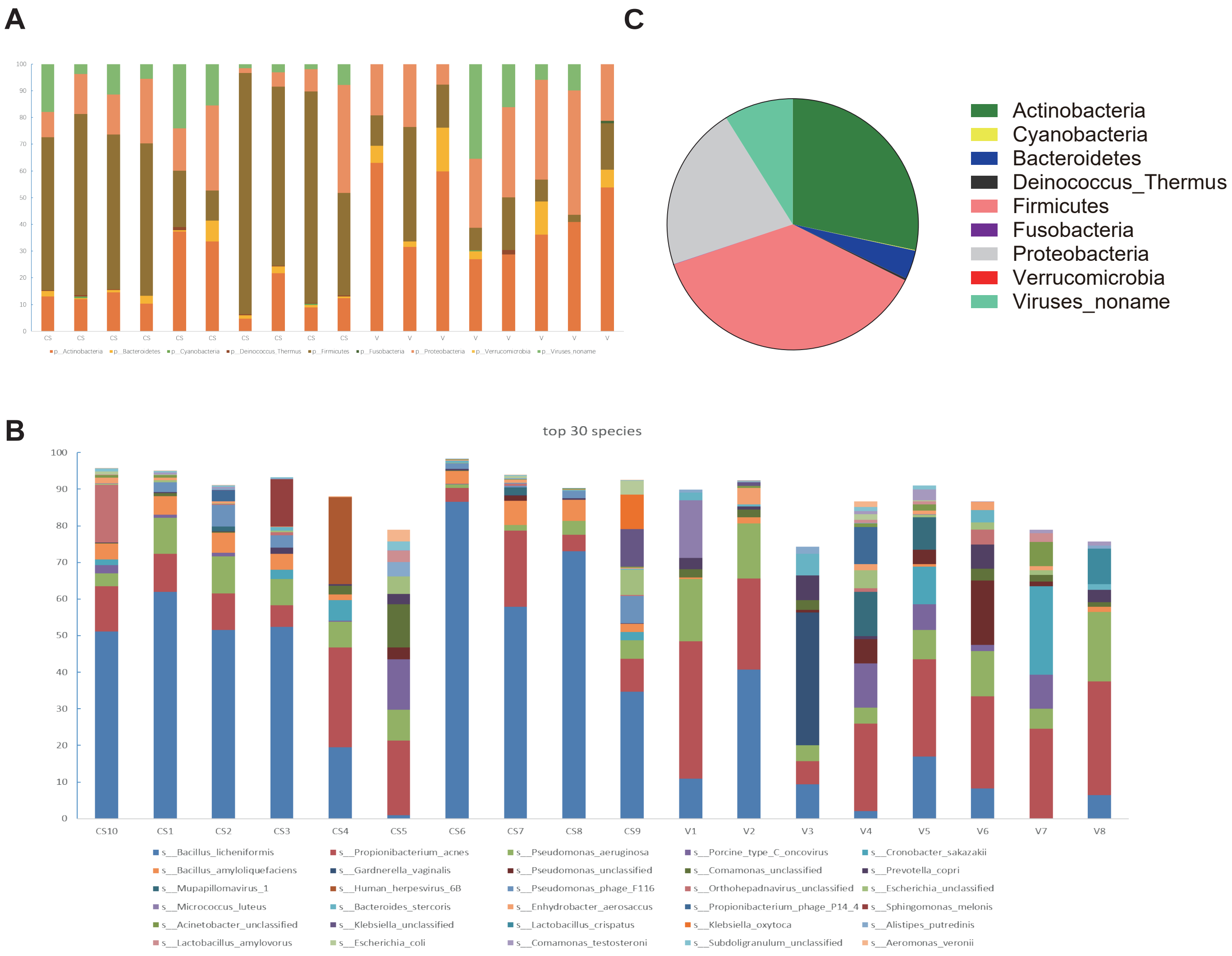

Supplement Figure 2

A

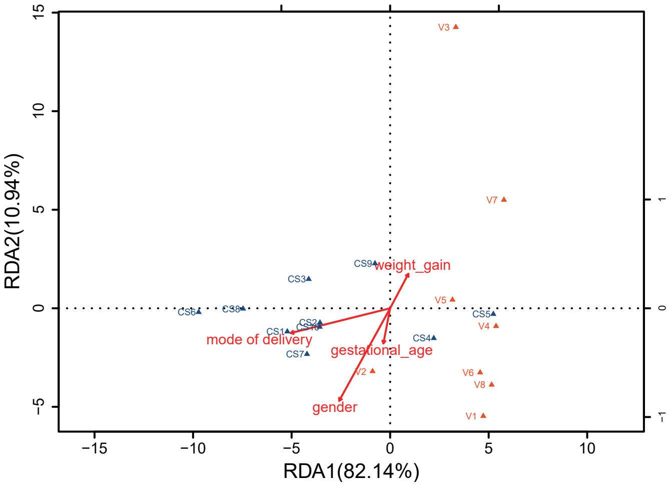

B

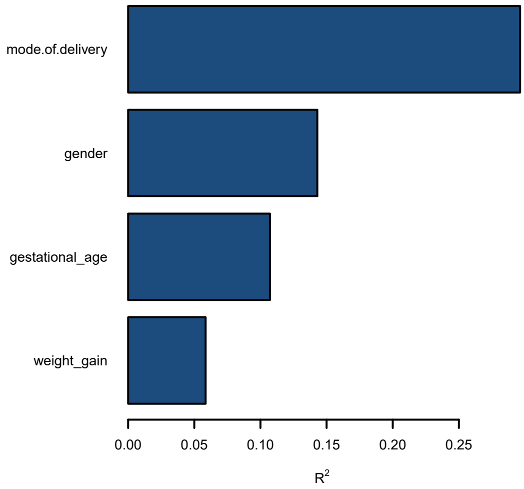

Supplement Figure 3

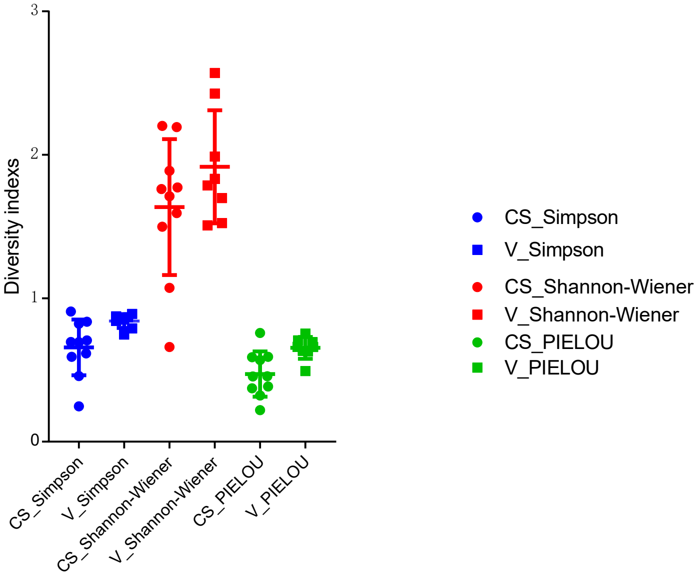

Supplement Figure 4

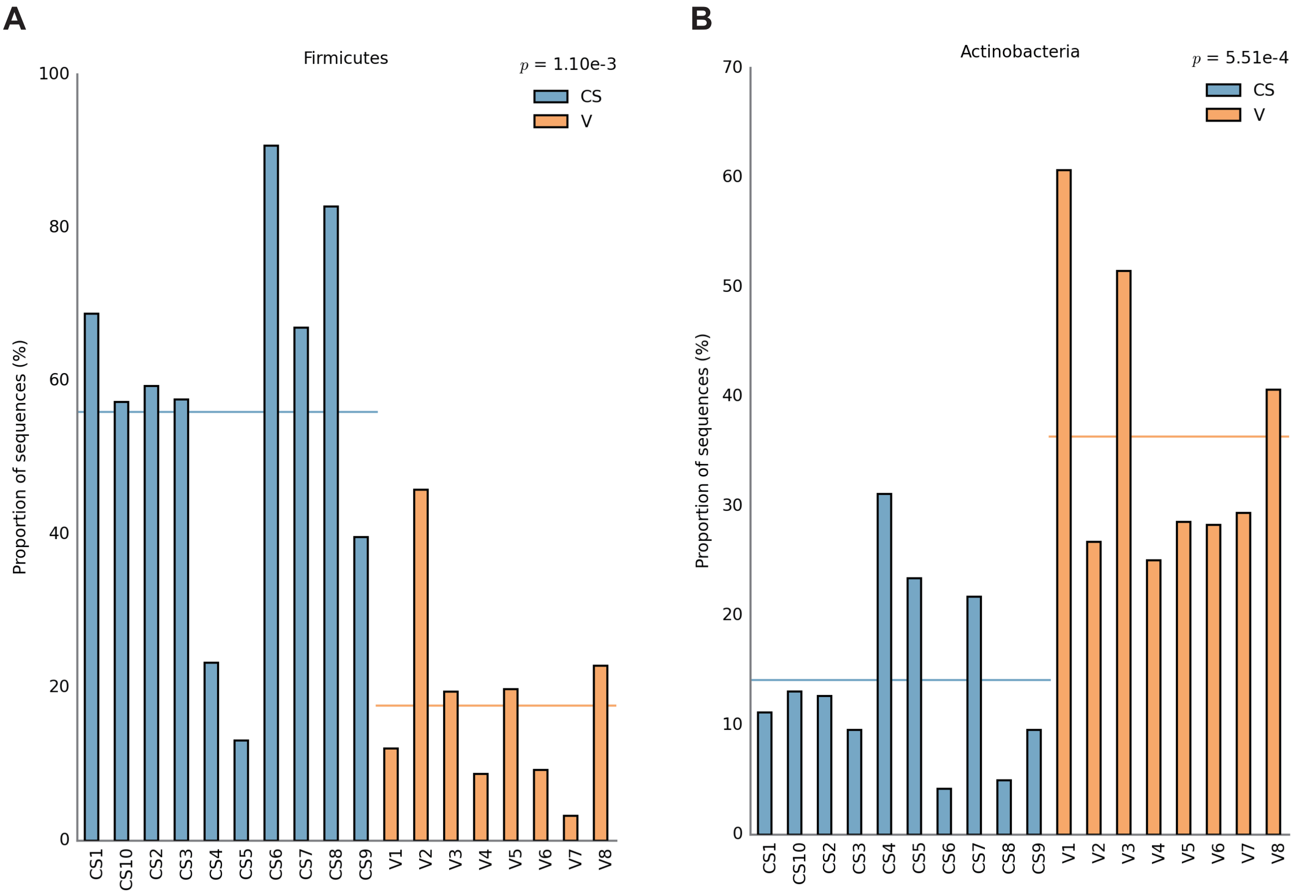

Supplement Figure 5

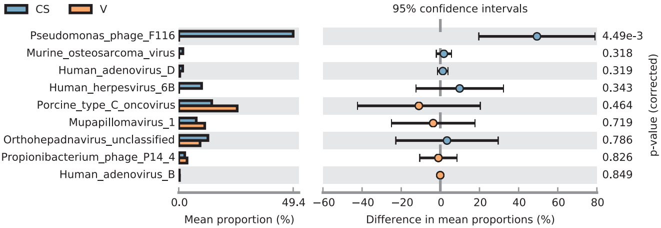

Supplement Figure 6

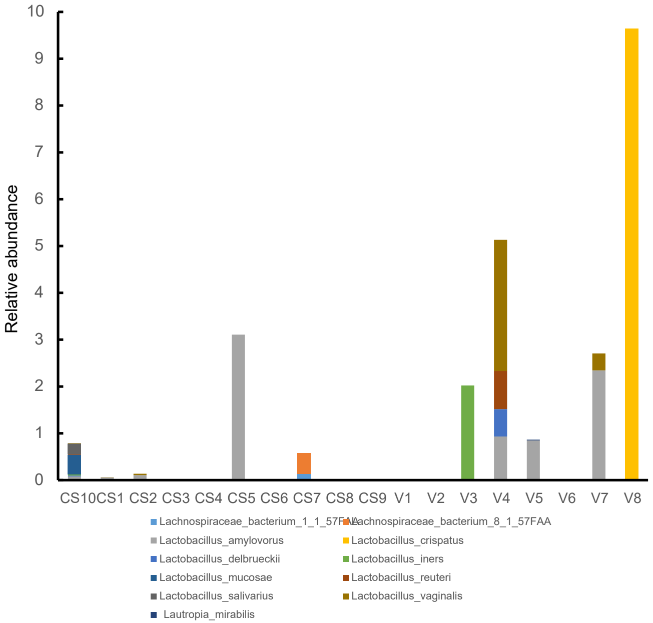

Supplement Figure 7

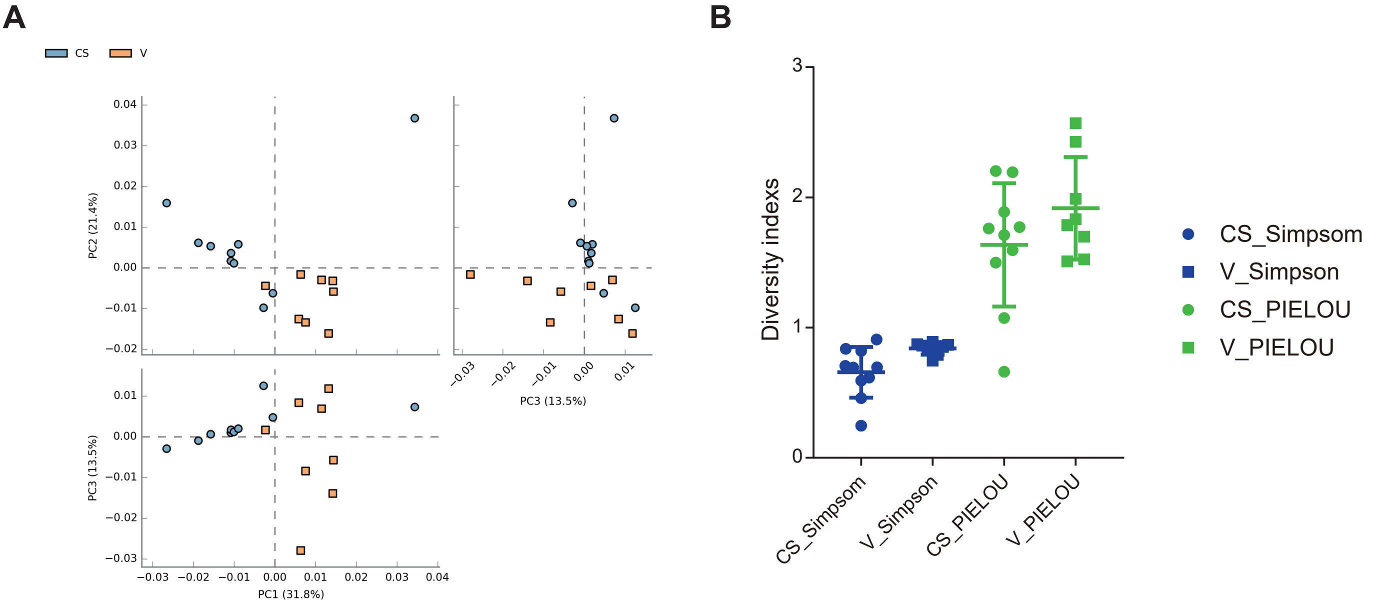

Supplement: Supplementary file 1 — Supplement Figure legends and table [file 41598_2018_21657_MOESM1_ESM.pdf]
